# Abaloparatide versus teriparatide: a head to head comparison of effects on fracture healing in mouse models

**DOI:** 10.1080/17453674.2018.1523771

**Published:** 2018-10-18

**Authors:** Magnus Bernhardsson, Per Aspenberg

**Affiliations:** Orthopaedics, Department of Clinical and Experimental Medicine, Faculty of Health Sciences, Linköping University, Linköping, Sweden

## Abstract

Background and purpose — Teriparatide accelerates fracture healing in animals and probably in man. Abaloparatide is a new drug with similar although not identical effects on the teriparatide receptor. Given at 4 times the teriparatide dose in a human osteoporosis trial, abaloparatide increased bone density more than teriparatide, and both reduced fracture risk. We investigated in mice whether abaloparatide stimulates fracture healing, and if it does so with the suggested dose effect relation (4:1).

Patients and methods — In a validated mouse model for metaphyseal healing (burr hole with screw pull-out), 96 mice were randomly allocated to 11 groups: control (saline), teriparatide or abaloparatide, where teriparatide and abaloparatide were given at 5 different doses each. In a femoral shaft osteotomy model, 24 mice were randomly allocated to 3 groups: control (saline), teriparatide (15 µg/kg) or abaloparatide (60 µg/kg). Each treatment was given daily via subcutaneous injections. Results were evaluated by mechanical testing and microCT.

Results — In the metaphyseal model, a dose-dependent increase in screw pull-out force could be seen. In a linear regression analysis (r = 0.78) each increase in ln(dose) by 1 (regardless of drug type) was associated with an increase in pull-out force by 1.50 N (SE 0.18) (p < 0.001). Changing drug from teriparatide to abaloparatide increased the force by 1.41 N (SE 0.60; p = 0.02).

In the diaphyseal model, the callus density was 23% (SD 10), 38% (SD 10), and 47% (SD 2) for control, for teriparatide and abaloparatide respectively. Both drugs were significantly different from controls (p = 0.001 and p = 0.008), but not from each other.

Interpretation — Both drugs improve fracture healing, but in these mouse models, the potency per µg of abaloparatide seems only 2.5 times that of teriparatide, rather than the 4:1 relation chosen in the clinical abaloparatide–teriparatide comparison trial.

Teriparatide is a truncated parathyroid hormone acting on the teriparatide receptor. It activates and stimulates osteoblasts and is an approved drug for osteoporosis. Teriparatide also stimulates fracture healing in a large number of animal models, and there are ample data to suggest it does also in humans, although a pivotal trial is missing. Teriparatide also activates osteoclasts, even though its anabolic effect on osteoblasts dominates its effects (Aspenberg and Johansson 2010, Aspenberg et al. 2016, Campbell et al. 2015).

Abaloparatide is a new peptide that also binds the teriparatide receptor with effects similar to teriparatide. Due to differences in details of this binding, osteoclast activation is described as less pronounced (Boyce et al. 2018).

In a large randomized trial comparing placebo, teriparatide, and abaloparatide in humans, the latter drug showed a stronger increase in bone density in the total hip region, the femoral neck, and the lumbar spine (Leder et al. 2015). Both drugs also reduced fracture risk. Abaloparatide was given daily at a dose of 80 µg and teriparatide at 20 µg. The choice of these doses was not motivated.

To our knowledge, no studies on abaloparatide and fracture healing have been published. This animal study addresses 2 questions: Does abaloparatide stimulate fracture healing similar to teriparatide, and does the dose relation 80 versus 20 µg represent a similar relation in potency?  

## Methods

### Overview

96 mice received bilateral drill holes in their proximal tibiae, where a steel screw was inserted in one of them. The mice received daily injections of either saline, teriparatide, or abaloparatide of different dosage (n = 8 per group). The mice were killed after 10 days and the tibias harvested. The pull-out force of the steel screw was measured using a material-testing machine and the bone formation in the drill hole was measured using microCT.

Another 24 mice received a mid-diaphyseal osteotomy in the femoral bone, stabilized by intramedullary nailing. The mice received a daily dose of either saline, teriparatide, or abaloparatide (n = 8 per group) for 28 days before they were killed. The material properties of the healed femur were measured by a 3-point bending test and the bone formation in the healing callus was measured using microCT.

### Animals

120 male C57BL/6 mice with a mean weight of 26 (SD 1.3) g were used. The animals were kept 4 per cage and given ad libitum access to food and water.

*Surgical procedure: tibia* — following procedure has been described elsewhere (Sandberg and Aspenberg 2015). The mice were anesthetized with isoflurane gas and received a subcutaneous injection of 0.2 mg/kg oxytetracycline as infection prophylaxis and 0.1 mg/kg buprenorphine for postoperative analgesia. Both hind legs were shaved and cleaned with chlorhexidine. Under aseptic conditions, a 5 mm incision was made below the knee, at the anterio-medial side of the tibia on both legs. The muscle covering the medial side of the tibia was scraped aside to expose the bone surface. A drill hole into the proximal tibia was made by hand, using a 0.4 mm diameter syringe needle approximately 1.0 mm below the growth plate. A custom-made screw (Ti6A14V grade 2, thread size M 0.7) was inserted into the hole in the right leg. The skin was then sutured.

*Surgical procedure: femur* — the following procedure has been described elsewhere (Sandberg and Aspenberg 2015). The preoperative preparation was the same as in the above section, with the difference that only the left leg was shaved and cleaned. A 7 mm longitudinal incision was made along the lateral thigh. The flexor and extensor muscles were separated bluntly, exposing the lateral femoral surface. The knee was opened laterally, and the patella was luxated medially. A 0.4 mm syringe needle was inserted as an intramedullary nail into the femur through the distal condyles. Before the needle was inserted, the tip of the needle was bent to act as a hook. Once inserted, the needle was rotated to fasten the hook into the proximal end of the femur, preventing the distal end of the needle from slipping out into the knee joint. The mid-femur was then exposed, and using a custom-designed pair of tongs the midshaft was cut. A suture was inserted to hold the patella in place before the skin was sutured.

### Drug treatment

The mice were randomized to different treatment groups after surgery. The tibia-mice were randomly allocated to 11 different groups: control (saline), teriparatide at the following doses: 0.56; 1.67; 5; 15; or 45 µg/kg. or abaloparatide at 2.2; 6.7; 20; 60; or 180 µg/kg. The femur-mice were randomly allocated to 3 different groups: control (saline), teriparatide (15 µg/kg), or abaloparatide (60 µg/kg). Treatment was administrated daily via subcutaneous injections in volumes of 0.03–0.05 mL.

The animals were killed by cervical dislocation after sedation with isoflurane. The tibiae or femur were harvested for further analysis.

### Mechanical testing

The following mechanical evaluations of tibia and femur has been described elsewhere (Sandberg and Aspenberg 2015). The mechanical properties of screw fixation and the femurs were analyzed using a computerized materials-testing machine (100R; DDL Inc., Eden Prairie, MN). The pull-out force of the screws was measured by a holder that was attached to the head of the screw and to the cross-head. The tibia was then fixed to the machine by a suture thread, looped around the bone next to the screw. The cross-head speed was 0.01 mm/s. Force at failure was recorded and defined as pull-out force. Stiffness was calculated by the machine after manual definition of the linear part of the load deformation curve, and energy was automatically calculated after a 10% drop from peak load.

For the femurs, the intramedullary needle was removed before the femur was mounted on a stand, with both ends resting with 6 mm between supporting points with the lateral aspect upwards. The central part of the callus was then pushed downwards by a cross-head, which was lowered with a speed of 0.05 mm/s. The force at failure, stiffness, and energy uptake were recorded as described above.

### microCT

The left tibiae, with a hole in the proximal metaphysis, and the femurs were analyzed with microCT (Skyscan 1174, v. 2; Bruker, Aarteselaar, Belgium). Topographic images of the bones with an isotropic voxel size of 11.2 μm were acquired at energy settings of 50 kV and 800 μA, using an aluminum filter of 0.25 mm, rotation step of 0.4°, and frame field averaging of 3 in a 180° scan. The images were reconstructed with NRecon (Skyscan, v. 1.6.8.0; Aarteselaar, Belgium) and corrected for ring artifacts and beam hardening.

For the drill holes, a volume of interest was defined as a cylinder with a diameter of 0.3 mm extending 1.0 mm into the bone marrow cavity, starting at the endosteal side of the cortical bone. For the femurs, a volume of interest was defined as a 2 mm long segment of the central callus, delineated manually, which excluded the old cortex and medullar cavity.

All definitions of volume of interest was done by a blinded examiner (MB), as was all other handling of the specimens.

Calibration of bone mineral density was carried out by scanning 2 hydroxyapatite phantoms of known density (0.25 and 0.75 g/cm^3^). Analysis of total bone volume (BV/TV) were performed in CTAn (Skyscan, v. 1.10; Aarteselaar).

### Statistics

The peak pull-out force of the tibial screw was pre-defined as the primary effect variable. In a linear regression analysis, pull-out force (N) was the dependent variable, and Ln(dose) (µg) and drug type were independent. Ln(dose) was a continuous variable, and drug was coded as 0 (teriparatide) or 1 (abaloparatide). The density (BV/TV) of the bone inside the burr hole was analyzed in the same way as the pull-out force.

**Figure:**
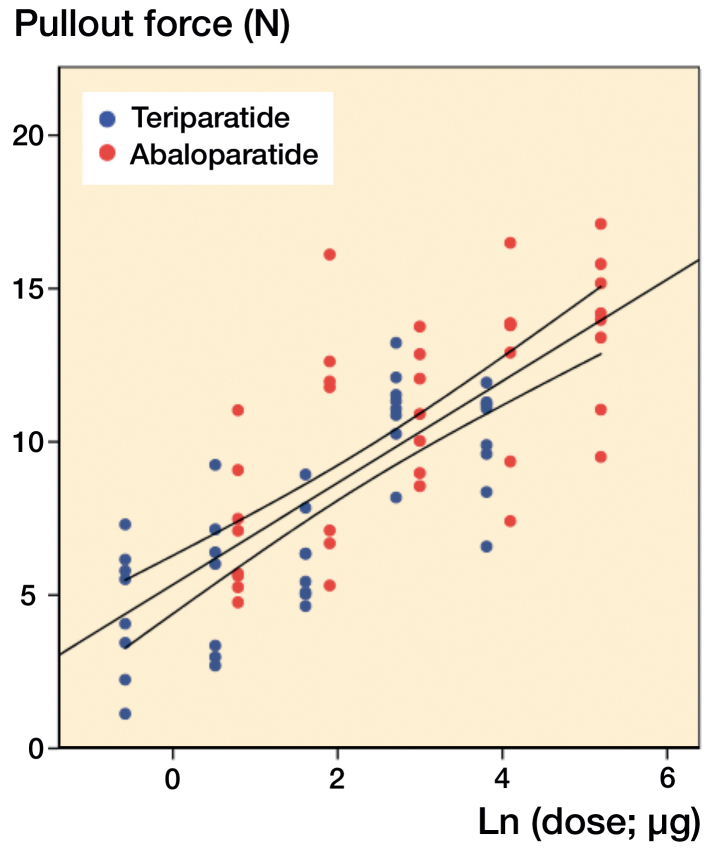
Linear regression of mechanical data from metaphyseal screws in tibiae, 10 days after insertion.

### Ethics, funding, and potential conflicts of interest

The study was approved by the Regional Ethics Committee for Animal Experiments and the animals were treated according to the institutional guidelines for care and treatment of laboratory animals. Funding was received from the Swedish Research Council (2031-47-5). Per Aspenberg has collaborated scientifically with Eli Lilly Corp, but received no grants or funding during the last 3 years. 

## Results

### Exclusions

In the metaphyseal model, 3 mice (1 control, 2 abaloparatide; 6.67 and 20 µg/kg) died postoperatively due to injuries from fighting amongst themselves. 2 screws (1 control and 1 teriparatide 1.67 µg/kg) fell out during harvesting before mechanical testing, probably due to poor insertion during surgery.

In the diaphyseal model, 2 specimens (1 control, 1 abaloparatide) were damaged during harvesting, before mechanical testing.

### Metaphyseal model

In the metaphyseal model, the control pull-out force was 5.8 (2.2) N. There was a dose-dependent increase up to 14 (SD 2.5) N at the highest dose of abaloparatide (Figure). In a linear regression analysis (r = 0.78) each increase in ln dose by 1 (regardless of drug type) was associated with an increase in pull-out force by 1.5 N (SE 0.18; p < 0.001). Changing drug from teriparatide to abaloparatide increased the force by 1.4 N (SE 0.60; p = 0.02). This corresponds to dose increase by a factor of 2.5.

**Table:** Results from mechanical and morphometric analysis of femoral shaft fractures after 28 days of healing. Values are mean (SD)

Group	Control	Teriparatide	Abaloparatide
	(n = 7)	(15 µg/kg; n = 8)	(60 µg/kg; n = 7)
Force at failure (N)	26 (11)	31 (15)	36 (11)
Stiffness (MPa)	20 (11)	27 (17)	27 (9.0)
Energy uptake (Nmm)	14 (6.9)	12 (3.6)	14 (4.6)
BV/TV (%)	23 (9.8)	38 (10)	47 (2.3)

Bone volume per total volume (BV/TV) in the tibial regions of interest showed slightly larger variation. In the linear regression analysis (r = 0.62), each increase in ln dose by 1 was associated with an increase in density by 10 percentage units (SE 1.7) regardless of drug type (p < 0.001). Changing drug from teriparatide to abaloparatide, regardless of dose, increased the density by 3 percentage units, but this was not statistically significant (p = 0.6).

### Diaphyseal model

In the diaphyseal model, confidence intervals were too large for meaningful interpretation of 3-point bending tests (Table), but the bone density (BV/TV) of the callus was 23% (SD 10), 38% (SD 10), and 47% (SD 2) for control, teriparatide, and abaloparatide respectively. Both drugs produced significantly denser callus than controls (p = 0.001 and p = 0.008), but they were not significantly different from each other (Table). 

## Discussion

Abaloparatide stimulated bone healing in both the diaphyseal and metaphyseal models. To our knowledge, abaloparatide has not been previously studied in a fracture-healing context.

The higher dose of abaloparatide versus teriparatide used by Ledeer et al. (2015) in the clinical comparison trial (4:1) only partly corresponded to a difference in potency (2.5:1) in our mouse models.

Our study has several weaknesses. Due to the assumption that abaloparatide would be 4 times more potent per µg, the highest dose was used only for this drug, and the lowest only for teriparatide in the metaphyseal model. The principal part of the study is the metaphyseal model, which has been thoroughly validated (Bernhardsson et al. 2015), while the diaphyseal model was used only as a complement. The pull-out force measurements provided the data with least random variation and comprised the predetermined primary outcome variable. The tibial density measurements support the mechanical results of a dose response but weaken the conclusion that abaloparatide is the more potent drug per µg. The mechanical evaluation of the diaphyseal model had large confidence intervals, while bone density measurements allowed the conclusions of a positive effect of both drugs also in diaphyseal healing.

It can be disputed whether screw pull-out really reflects cancellous bone fracture healing. It is, however, clear that the force reflects the strength of the bone that has regenerated and grasped the screw threads during the 10 days after screw insertion, so it can always be stated that the force is a measure of local cancellous bone formation in response to trauma. This is at least very similar to cancellous bone healing (Bernhardsson et al. 2015).

We are unaware of species differences in receptor binding characteristics, which could be different between mouse and man. Such differences might explain the differences in dose relations between the species.

Even though this was a mouse study of fracture healing, it is tempting to speculate about the interpretation of the clinical abaloparatide teriparatide comparison trial (Leder et al. 2015). Possibly, the difference in effect between abaloparatide and teriparatide in this clinical trial partly reflects that abaloparatide was given at a higher dose, and that a moderate dose reduction would have eliminated the difference. This possibility is supported by the fact that the abaloparatide group also had more treatment discontinuations due to mild side effects, such as nausea, than teriparatide and placebo.

In conclusion, abaloparatide and teriparatide stimulated bone regeneration in mouse fracture models to a similar extent.  

Both authors were involved in designing the study, analyzing the data, preparing and approving the submitted manuscript. MB conducted the experiments and acquired the data. 

*Acta* thanks Ming Ding and Sten Rasmussen for help with peer review of this study.
